# The Association Between Caregiving Burden and Depressive Symptoms Among U.S. Chinese Adult Children

**DOI:** 10.1093/geroni/igaa057.1158

**Published:** 2020-12-16

**Authors:** Shuting Liang, XinQi Dong

**Affiliations:** 1 Rutgers University–New Brunswick, Chicago, Illinois, United States; 2 Rutgers University, New Brunswick, New Jersey, United States

## Abstract

Existing research has showed the impact of caregiving burden on physical and psychological outcomes among adult children, but less have examined its association among Chinese immigrants in the US. This research will present the association between caregiving burden and depressive symptoms among U.S. Chinese adult children. Cross-sectional data were drawn from the PIETY study with 547 Chinese adult children aged over 21 years old in the greater Chicago area between 2012-2014. Caregiving burden was assessed by 24-item caregiver burden developed by Novak and Guest and is composed of five factors: time-dependence, developmental, physical, social, and emotional burden. Depressive symptoms were assessed by the nine-item Patient Health Questionnaire. Logistic regression analysis was conducted. In our sample, 241 (44%) adult children had depressive symptoms and 174 (72.2%) were female. In the result of multivariate analysis, after adjusting for covariates, developmental burden (Odds ratio [OR] 1.13 [1.05-1.21]), physical burden (OR 1.17 [1.06-1.28]), social burden (OR 1.20 [1.08-1.32]), and emotional burden (OR 1.22 [1.11-1.35]) were positively associated with reporting any depressive symptoms. However, the time-dependence burden was not associated with depressive symptoms. The findings highlight the potential impact of caregiving burden on depressive symptoms and how different domains of caregiving burden are associated with depressive symptoms among Chinese caregivers in the U.S. Future research should include multidimensional social supports or acculturation as underlying factors which might affect the relationship between caregiver burden and depressive symptoms across Chinese community in the US.

